# Nivel de ansiedad y respuesta fisiológica ante tratamientos dentales invasivos. un estudio longitudinal

**DOI:** 10.21142/2523-2754-1104-2023-175

**Published:** 2023-12-28

**Authors:** Fernando Jesús Arellano-Cabezas, Kilder Maynor Carranza-Samanez

**Affiliations:** 1 Carrera de Estomatología, Universidad Científica del Sur. Lima, Perú. 100036527@cientifica.edu.pe Universidad Científica del Sur Carrera de Estomatología Universidad Científica del Sur Lima Peru 100036527@cientifica.edu.pe; 2 Research Group in Dental Sciences, Carrera de Estomatología, Universidad Científica del Sur. Lima, Perúkcarranza@cientifica.edu.pe Universidad Científica del Sur Research Group in Dental Sciences Carrera de Estomatología Universidad Científica del Sur Lima Peru kcarranza@cientifica.edu.pe

**Keywords:** ansiedad al tratamiento odontológico, presión arterial, saturación de oxígeno, pulso arterial, anestésicos locales, dental anxiety, arterial pressure, oxygen saturation, pulse, local anesthetics

## Abstract

**Objetivo:**

Determinar la relación entre el nivel de ansiedad y la respuesta fisiológica ante tratamientos dentales invasivos.

**Materiales y métodos:**

: Se utilizó una muestra por conveniencia de 180 pacientes (73 varones y 107 mujeres), entre 18 y 58 años, en tratamiento con anestésicos locales de tres consultorios dentales privados de la ciudad de Lima. Se realizaron mediciones antes, durante y después del tratamiento sobre ansiedad, según el cuestionario IDARE de 40 preguntas (20 de rasgo y 20 de estado), y se evaluó su respuesta fisiológica según saturación de oxígeno (SO), pulso arterial y presión arterial (PA), medidos con pulsioxímetro y tensiómetro digitales. Las pruebas de Friedman y correlación de Spearman fueron utilizadas y se trabajó con un valor de p < 0,05.

**Resultados:**

La mayoría de los pacientes tuvo un nivel de ansiedad medio antes del tratamiento dental (estado: 49,4 % y rasgo: 55,6%). La SO, el pulso y la PA aumentaron luego de la aplicación del anestésico y disminuyeron al final del tratamiento, con diferencias significativas (p < 0,05). Los puntajes de ansiedad se correlacionaron significativamente solo con el pulso (estado: r = 0,238-0,564; rasgo: r = 0,174-0,323) y la PA (estado: r = 0,429-0,699; rasgo: r = 0,312-0,465) (p < 0,05).

**Conclusión:**

La ansiedad estado-rasgo tuvo una relación positiva con las dimensiones fisiológicas de presión arterial y pulso ante el tratamiento dental con anestésicos locales.

## Abstract (en)

**Objective:**

To determine the relationship between the level of anxiety and the physiological response to invasive dental treatments.

**Materials and methods:**

A convenience sample of 180 patients was formed (73 men and 107 women) ≥18 years (range: 18-58) undergoing treatment with local anesthetics from three private dental offices in the city of Lima. Measurements were made before, during and after treatment on anxiety according to the IDARE questionnaire of 40 questions (20 trait and 20 state) and their physiological response was evaluated according to oxygen saturation (OS), pulse and blood pressure (BP) measured. with digital pulse oximeter and blood pressure monitor. The Friedman and Spearman correlation tests were used, working with a value of P<0.05.

**Results:**

Most patients had a medium level of anxiety before dental treatment (state 49.4% and trait 55.6%). OS, pulse and BP increased after application of the anesthetic and decreased at the end of treatment, with significant differences (*P*<0.05). Anxiety scores were significantly correlated only with pulse (state: r=0.238-0.564; trait: r=0.174-0.323) and BP (state: r=0.429-0.699; trait: r=0.312-0.465) (*P*<0.05).

**Conclusion:**

State-trait anxiety had a positive relationship with the physiological dimensions of blood pressure and pulse before dental treatment with local anesthetics.

## INTRODUCCIÓN

La ansiedad puede ser comprendida como un rasgo de la personalidad, lo que se denomina ansiedad-rasgo. Esta condición latente puede agudizarse ante determinadas situaciones, y ser temporal y fluctuante al tiempo ^(^^1^^,^^2^, lo que se considera como ansiedad-estado ^3^. La ansiedad tiene diversos signos y síntomas, como dilatación arterial, cefalalgia, transpiración, entre otros ^4^.

En el área odontológica, una mala experiencia dental de joven puede agravar el nivel de ansiedad en los procedimientos. Ello podría presentarse por un tratamiento prolongado, el uso de instrumental pulsante o la emisión de ruidos y vibraciones ^5^. Los tratamientos odontológicos más relacionados con estos sucesos son las terapias invasivas y prolongadas, como los tratamientos endodónticos y las cirugías maxilofaciales ^6^^,^^7^.

Los autoinformes se emplean con mayor frecuencia para evaluar la ansiedad. El más conocido es la escala de ansiedad dental modificada de Humphries ^8^; sin embargo, su adaptación al contexto cultural de países hispanohablantes es limitada. El inventario de ansiedad estado-rasgo (IDARE) ^9^ es otro instrumento adaptado de la versión en inglés State Trait - Anxiety Inventary (STAI). Esta herramienta también es muy utilizada en múltiples idiomas y ha sido adaptada al contexto por psicólogos peruanos ^10^.

La ansiedad se relaciona con las respuestas fisiológicas y puede ser cuantificada ^11^. Esta activa la rama simpática del sistema nervioso autónomo y se manifiesta como respuesta de huida o lucha frente a alguna situación. Ello involucra modificaciones en diferentes sistemas, como el respiratorio y el cardiovascular, con el incremento en la frecuencia respiratoria, lo que reduce la presión parcial de CO2, y produce en el corazón el aumento de la frecuencia cardiaca y su potencial de llevar oxígeno a los tejidos ^12^.

La ansiedad genera cambios en los parámetros fisiológicos, por lo que resulta relevante cuantificarla en entornos clínicos. Entre estos destacan la saturación de oxígeno (SO) y el pulso, que pueden ser medidos con un oxímetro, ^13^ y la presión arterial (PA), que utiliza el esfigmomanómetro para su medición ^14^. Estudios previos hallaron una relación entre la ansiedad del paciente y el nivel de FC en tratamientos dentales ^15^^-^^17^.

Si bien existe un sustento fisiológico que relaciona respuestas ante estímulos ansiosos, la evidencia aplicada a lo largo de la atención odontológica es limitada y heterogénea por la diversidad de parámetros fisiológicos y metodologías de medición de ansiedad. Por ello, el propósito de este estudio fue relacionar la ansiedad con la respuesta fisiológica ante tratamientos odontológicos invasivos en un contexto de pacientes peruanos. 

## MATERIALES Y MÉTODOS

Esta investigación observacional longitudinal fue aprobada por el Comité Institucional de Ética de Investigación (CIEI) de la Universidad Científica del Sur (N.° 125-CIEI-CIENTÍFICA-2022). El estudio siguió las directrices de la Declaración de Helsinki y obtuvo consentimientos informados de los participantes. Se conformó una muestra a conveniencia de 180 pacientes adultos atendidos en tres consultorios privados de Lima en 2022. Los criterios de inclusión fueron: pacientes voluntarios, ≥ 18 años, con la clasificación del estado físico ASA I y cuya atención incluyera tratamientos invasivos.

El cuestionario IDARE ^18^ fue aplicado en su versión en español adaptada y validada para medir ansiedad dental ^19^. El instrumento tuvo 40 ítems con 4 respuestas tipo Likert: (1 = no en absoluto, 2 = un poco, 3 = bastante, y 4 = mucho). Los primeros 20 ítems midieron la dimensión ansiedad-rasgo (D1: cómo se siente habitualmente) y los siguientes 20 evaluaron la dimensión ansiedad-estado (D2: cómo se siente ahora mismo, en estos momentos). Los ítems con valoración inversa fueron 10 en D1 (ítems 1, 2, 5, 8, 10, 11, 15, 16, 19 y 20) y 7 en D2 (ítems 21, 26, 27, 30, 33, 35 y 39). La puntuación de ansiedad osciló entre 20 y 80 puntos por cada dimensión, y se categorizó en tres niveles: bajo (≤30), medio (31-44) y alto (≥45). Una mayor puntuación indicó mayor ansiedad dental. 

Un mismo examinador entrenado (FJAC) evaluó a los pacientes que acudieron a los consultorios mediante la encuesta IDARE, y midió la SO (SpO2: saturación de oxígeno en sangre) y pulso arterial (lpm: latidos por minuto) con un pulsioxímetro digital (Choicemmed), la presión arterial sistólica (PAS) al primer latido del corazón y la diastólica (PAD) cuando dejó de escuchar el latido con el brazalete medidor de presión digital en mmHg (Riester 1350). 

Todas las variables se midieron en tres momentos: antes, durante y después del tratamiento dental que involucró uso de anestésicos locales con epinefrina. La primera medida se realizó en el sillón dental previo al tratamiento, la segunda se aplicó después de la aplicación del anestésico y tras la confirmación del odontólogo del efecto en el paciente, y la tercera se registró en el sillón dental al concluir el tratamiento. 

La estadística descriptiva incluyó mediana (Me), rango intercuartil (RQI), frecuencias y porcentajes. La prueba de Kolmogorov-Smirnov corroboró la no normalidad, por lo que se utilizó pruebas no paramétricas de Friedman con *post-hoc* por parejas y correlación de Spearman. Se valoró de la siguiente manera: nula (0), muy baja (0,01-0,19), baja (0,2-0,39), moderada (0,4-0,59), alta (0,6-0,79), muy alta (0,8-0,99) y perfecta ^1^. La data se analizó con el programa estadístico SPSS versión 25, con un nivel de significación de 0,05.

## RESULTADOS

La mayoría de las participantes eran mujeres (59,4 %) con residencia en el distrito de Chorrillos (70,0 %). Las edades oscilaron de 18 y 58 años, con una edad media de 35,00 ± 4,65 años (Tabla 1). El puntaje D1-estado fue mayor (Me = 35,5 [RQI = 17]) que en D2-rasgo (Me = 32 [RQI = 10]). La mayoría de las participantes tuvieron una ansiedad media basal en ambas dimensiones (49,4 % y 55,6 %, respectivamente) (Tabla 2). Los rangos oscilaron, para SO, de 94 a 100; para pulso, de 54 a 115; para PAD, de 57 a 87; y para PAS, de 101 a 134. Todos los parámetros fisiológicos aumentaron significativamente luego de la aplicación del anestésico (SO↑2, pulso↑6, PAD↑8 y PAS↑7) y, posteriormente, disminuyeron significativamente al concluir el tratamiento (SO ↓1, pulso ↓4, PAD↓7 y PAS↓3) (*P*=0,00) (Tabla 3). El puntaje de ansiedad-estado tuvo una correlación positiva altamente significativa solo con el pulso de forma baja-moderada (estado: r = 0,238-0,564) y con la PA de forma moderada-alta (estado: r = 0,429-0,699) (p < 0,001). Por su parte, la ansiedad-rasgo se correlacionó de forma positiva significativa solo con el pulso de forma muy baja-baja (rasgo: r = 0,174-0,323) y con la PA de forma baja-moderada (rasgo: r = 0,312-0,465) (p < 0,05) (Tabla 4).

**Tabla 1:** Características demográficas de los participantes

Características demográficas		n (%)
Sexo	Masculino	73 (40,6%)
	Femenino	107 (59,4%)
Edad (años)	Rango	18-58
	Media ± DS	35,00 ± 4,65
Total		180 (100,0%)

**Tabla 2:** Puntajes y niveles según tipos de ansiedad estado-rasgo medidos antes del tratamiento

Puntaje y nivel	Ansiedad-estado	Ansiedad-rasgo
Puntaje (Mediana [RIQ])	35,5 [17]	32 [10]
Nivel, n (%)		
Baja (≤30 puntos)	51 (28,3%)	65 (36,1%)
Media (31-44 puntos)	89 (49,4%)	100 (55,6%)
Alta (≥45 puntos)	40 (22,2%)	15 (8,3%)

Me, mediana. RQI, rango intercuartil.

**Tabla 3:** Comparación de los valores de respuesta fisiológica entre los tres tiempos de tratamiento

Parámetros de respuesta fisiológica	Tiempos de medición, mediana [RIQ]			p valor
	Antes	Durante	Después	
Saturación de oxígeno (%)	97 [2]a	98 [1]b	97 [1]c	< 0,001*
Pulso (ppm)	73 [17]a	79 [15]b	75 [14]c	< 0,001*
Presión arterial diastólica (mmHg)	66 [5]a	74[6]b	67 [6]c	< 0,001*
Presión arterial sistólica (mmHg)	112,5 [8]a	119 [9]b	116 [8]c	< 0,001*

ppm: Pulsaciones por minuto. Letras distintas en cada fila indican diferencias entre tiempos de medición. Prueba de Friedman con análisis *post-hoc* por parejas. *p < 0,05.

**Tabla 4:** Correlación del puntaje de ansiedad y respuesta fisiológica en los diferentes tiempos de tratamiento

Parámetros de respuesta fisiológica	Puntaje de ansiedad, rho (p valor)		
	Antes	Durante	Después
Según estado			
Saturación de oxígeno	0,006 (0,941)	0,142 (0,570)	-0,011 (0,888)
Pulso	0,301 (<0,001*)	0,564 (<0,001*)	0,238 (<0,001*)
Presión arterial	0,429 (<0,001*)	0,699 (<0,001*)	0,625 (<0,001*)
Según rasgo			
Saturación de oxígeno	-0,64 (0,394)	0,177 (0,170)	-0,061 (0,412)
Pulso	0,174 (0,019*)	0,323 (<0,001*)	0,193 (0,010*)
Presión arterial	0,312 (<0,001*)	0,465 (<0,001*)	0,334 (<0,001*)

rho: coeficiente de correlación de Spearman. *p < 0,05

## DISCUSIÓN

El objetivo del presente estudio tuvo como objetivo relacionar la ansiedad con la respuesta fisiológica ante tratamientos odontológicos invasivos. Según los resultados del estudio, se halló que los puntajes de ansiedad se correlacionaron significativamente de forma positiva con el pulso y la PA, pero no con SO. De esta manera, se corroboró parcialmente la hipótesis de correlación de las variables.

En este estudio, se halló que la correlación fue significativa de la PA con la ansiedad estado de forma moderada-alta y con ansiedad rasgo de forma baja-moderada. Dantas *et al*. ^20^ evaluaron la influencia de la ansiedad ante extracción de terceros molares sobre sus signos vitales (SO, FC y PAS/PAD) en diferentes momentos, y encontraron que la ansiedad influyó únicamente en el aumento de la PA. A diferencia de este estudio, ellos evaluaron tratamientos más invasivos y probaron distintos tipos de anestésicos.

Otro hallazgo de este estudio fue una correlación significativa entre el pulso y la ansiedad-estado de forma baja-moderada y con ansiedad rasgo de forma muy baja-baja. También Gil-Abando ^16^ estudió varios parámetros fisiológicos y encontró una correlación leve entre la ansiedad y el pulso. Este resultado puede deberse a que se aplicó ante tratamientos no invasivos en los que la respuesta fisiológica sería menos intensa. 

Según Olivieri *et al*. ^21^, en su estudio de evaluación de la ansiedad en pacientes monitorizando la FC y SO antes, durante y después de un tratamiento de conductos, al igual que el presente estudio, no hallaron diferencias clínicas de la SO respecto de la ansiedad en los distintos momentos de medición. 

De acuerdo con el inventario IDARE, se obtuvo un nivel medio de ansiedad-estado y la ansiedad-rasgo basal en el 49,4% y el 55,6%, respectivamente, mientras que casi un 37% presentaron nivel bajo. Gurran *et al*. ^22^ obtuvieron resultados distintos con niveles de ansiedad alta en el 41,2% utilizando el STAI, la DAS y la escala de calificación numérica. La diferencia podría deberse a que, en ese estudio, los pacientes fueron sometidos a tratamientos más invasivos, como la extracción de terceras molares, lo que predispondría a niveles mayores de ansiedad. Sirian *et al*. ^23^ hallaron que altos valores de ansiedad rasgo-estado se correlacionaron con las experiencias dentales desagradables previas informadas por los pacientes sometidos a cirugía del tercer molar. Este resultado es compatible con un escenario de tratamientos más traumáticos e invasivos. 

En esta investigación se halló un patrón de todos los parámetros fisiológicos (SO, pulso y PA) donde hubo un aumento luego de la aplicación del anestésico, y una posterior disminución al final del tratamiento. Alemany *et al*. ^24^ determinaron cambios hemodinámicos durante el tratamiento de extracción de molares, donde el pulso y la PA mostraron cambios significativos, a diferencia de la SO, que se mantuvo constante. Impijn *et al*. ^25^ evaluaron la PA y la FC durante el tratamiento dental y hallaron que 16 de 40 pacientes presentaron taquicardia. Ello fue distinto a lo obtenido en nuestro estudio, en el cual, a pesar del aumento de las pulsaciones durante el tratamiento, la mayoría de los pacientes se encontraron en rangos normales. Brand ^26^ encontró un aumento significativo en la PA ante el tratamiento dental, al igual que este estudio. Brand *et al*. ^27^ determinaron que, de varias respuestas fisiológicas, solo la PA registró un aumento ante tratamientos restauradores sin anestesia local y de extracción dental. Podría considerarse que el dolor experimentado por el paciente influyó en el aumento de la PA. 

Según la literatura, tanto la hipertensión como la taquicardia pueden ser resultado de la liberación de catecolaminas, ya que su acción cardiaca aumenta la frecuencia en que se contrae el miocardio, lo que provoca un aumento del trabajo cardíaco ^28^^,^^29^. Asimismo, tienen acción predominante sobre el lecho vascular sistémico con un aumento de la resistencia periférica que provoca la hipertensión arterial. La liberación de las catecolaminas está inducida por aspectos como la ansiedad, el estrés o la actividad física ^30^. Esto podría explicar nuestros resultados de correlación entre ansiedad y respuesta fisiológica, donde los mayores valores se produjeron ante el procedimiento dental.

Las limitaciones del estudio fueron no poder controlar las condiciones clínicas aplicadas a criterio del odontólogo, tales como los tipos de anestésicos, la cantidad de cartuchos y el tipo de procedimiento. A pesar de que el instrumento de ansiedad aplicado se utiliza en múltiples países, puede tener limitación en la fidelidad y veracidad de los datos dados por el paciente. Debido a la elección a conveniencia de la muestra, no es posible generalizar los resultados.

## CONCLUSIONES

El nivel de ansiedad se relacionó de forma positiva con la presión arterial y el pulso ante tratamientos dentales invasivos con anestésicos locales. La mayoría de las pacientes presentaron un nivel medio de ansiedad antes del tratamiento dental, lo que aumentó sus valores de saturación de oxígeno, pulso y presión arterial luego de recibir anestésicos locales.

## Fuente de financiamiento:

Autofinanciado
